# Individual and contextual level enablers and barriers determining electronic community health information system implementation in northwest Ethiopia

**DOI:** 10.1186/s12913-023-09629-8

**Published:** 2023-06-16

**Authors:** Tesfahun Hailemariam, Asmamaw Atnafu, Lemma Derseh Gezie, Jens Johan Kaasbøll, Jörn Klein, Binyam Tilahun

**Affiliations:** 1 Department of Health Informatics, Institute of Public Health, College of Medicine and Health Sciences, University of Gondar, Gondar, Ethiopia; 2 Department of Health Informatics, College of Health Sciences, Hawassa, Ethiopia; 3 Department of Health System and Policy, Institute of Public Health, College of Medicine and Health Sciences, University of Gondar, Gondar, Ethiopia; 4 Department of Epidemiology and Biostatistics, Institute of Public Health, College of Medicine and Health Sciences, University of Gondar, Gondar, Ethiopia; 5 Department of Informatics, University of Oslo, Oslo, Norway; 6 Department of Nursing and Health Sciences Campus Porsgrunn, University of South-Eastern Norway, Porsgrunn, Norway

**Keywords:** eCHIS, Implementation, Health data quality, Use, Service provision, CFIR framework, HEWS, Wogera, Northwest, Ethiopia

## Abstract

**Background:**

The government of Ethiopia has envisioned digitalizing primary healthcare units through the electronic community health information system (eCHIS) program as a re-engineering strategy aiming to improve healthcare data quality, use, and service provision. The eCHIS is intended as a community-wide initiative to integrate lower health structure with higher administrative health and service delivery unit with the ultimate goal of improving community health. However, the success or failure of the program depends on the level of identifying enablers and barriers of the implementation. Therefore, this study aimed to explore individual and contextual-level enablers and barriers determining eCHIS implementation.

**Method:**

We conducted an exploratory study to determine the enablers and barriers to successfully implementing eCHIS in rural Wogera district, northwest Ethiopia. In-depth interviews and key informant interviews were applied at participants from multiple sites. A thematic content analysis was conducted based on the key themes reported. We applied the five components of consolidated framework for implementation research to interpret the findings.

**Results:**

First, based on the intervention's characteristics, implementers valued the eCHIS program. However, its implementation was impacted by the heavy workload, limited or absent network and electricity. Outer-setting challenges were staff turnover, presence of competing projects, and lack of incentive mechanisms. In terms of the inner setting, lack of institutionalization and ownership were mentioned as barriers to the implementation. Resource allocation, community mobilization, leaders’ engagement, and availability of help desk need emphasis for a better achievement. With regard to characteristics of the individuals, limited digital literacy, older age, lack of peer-to-peer support, and limited self-expectancy posed challenges to the implementation. Finally, the importance of mentoring and engaging community and religious leaders, volunteers, having defined plan and regular meetings were identified elements of the implementation process and need emphasis.

**Conclusion:**

The findings underlined the potential enablers and barriers of eCHIS program for quality health data generation, use, and service provision and highlighted areas that require emphasis for further scale-up. The success and sustainability of the eCHIS require ongoing government commitment, sufficient resource allocation, institutionalization, capacity building, communication, planning, monitoring, and evaluation.

**Supplementary Information:**

The online version contains supplementary material available at 10.1186/s12913-023-09629-8.

## Background

The right data, information, and decision-making are fundamentals, but they are challenged by several factors [1]. In most developing countries health reports related to manual health data management practices, are incomplete and inaccurate [2]. Safe and reliable healthcare is also challenged due to poor quality of health data production and use [3]. In the health sector, technical, behavioral, and organizational characteristics are hindering factors for the success of health information systems [1].

Globally, access to essential healthcare services is low due to a shortage of health workers [4]. Training community health workers(CHWs) has been a solution to fill the gaps in healthcare accessibility [4]. According to the World Health Organization (WHO), investing on health information systems (HIS) improves health service quality [5, 6] through enhanced data quality [5] and decision-making [7].

In Ethiopia, the health management information system (HMIS) has been implemented to ensure quality data production and use [8]. Information revolution (IR) is one of the four transformation agenda of the current health sector transformation plan (HSTP) [9]. Ethiopia has been implementing the health extension program (HEP) since 2003, comprising female health extension workers (HEWs) [10]. They are tasked with providing maternal and child health services at primary health care units (PHCUs) [11]. They bridge health systems, households and perform home visits, assessments, treatment of diseases, routine health post activities, and outreach related activities in the community [12].

The health system leaders access and use the reports of HEWs for planning and decision making [13, 14]. Even though health posts provide data for planning and decision-making, the quality of the data is poor [3] and the figures on key indicators from PHCUs are also subjected to errors [15]. The limited capacity of HEWs, low educational preparedness, workload, exhaustive, and time-consuming manual data handling approaches contribute to poor data quality and use [16, 17].

The implementation of digital solutions have significant benefits in various healthcare settings by lowering healthcare costs, advancing access to information, enabling quick access to clients’ records, and improving communication between clients and healthcare providers [18–20]. Moreover, it has become a solution to improve health service delivery processes and health outcomes [21–23] and contributed in improvements of maternal healthcare [24], quality in health service delivery and adherence in health service utilization [25], health behavioral change [26], and health data quality and use [27]. Moreover, using digital health solutions has paramount benefits in improving CHWs performance [28, 29], reducing errors [30], increasing motivation [31], and creating learning opportunities [31].

The digital tool, eCHIS, was designed to capture health data, use, and provide healthcare to patients or clients. However, it is known that individual, technological, organizational, and social factors contribute to success or failure of digital health solutions [20]. For example, participants who were grouped as having a young age [32] and good educational level were enablers of the digitization [32]. Moreover, at one hand, perceived enjoyment [33], favorable attitudes [34–36] and good habits [37] were mainly enabling factors of digital health implementation. On the other hand, anxiety or fear of new technologies [20, 38] and older age, lack of interest, negative perceptions of effort improvement and low knowledge sharing practices are factors affecting digital health implementation [38].

From a technological factors perspective, positive effort expectancy [32–40], performance expectancy [34, 37–41], having computer literacy, long duration of mobile device use [32] have the potential to enable digital health implementation; however, unfamiliarity with the system and not ease of use constraint the implementation [38]. Furthermore, organizations with facilitating conditions [33, 34, 37–39] and good culture [38] were enabling factors, whereas lack of infrastructure, lack of system training, and lack of leadership engagement were significant barriers to digital health implementation in resource-limited settings [38]. Moreover, social influence has a positive impact on digital health implementation [38–41].

There is a global movement towards the integration of community health into the broader health system and using CHWs as a key strategy to achieve universal health coverage (UHC) [42]. Many developing countries have implemented a variety of primary health services programs and in most of them CHWs have fulfilled generalist health functions [43]. Since the inception of health policy in Ethiopia, the government of Ethiopia has invested a lot in community health information system (CHIS) using different approaches. One of the proven approaches is the HEP program for improving community health. According to an Ethiopian national report, health posts at the community level are available in more than 97% of kebeles and most of them are staffed with at least two female HEWs [43]. Due to low HEWs performances and a strong need to improve data quality, use, and service provision, the government of Ethiopia is committed in optimizing the HEP program so as to realize UHC through primary healthcare improvement [43]. Digitization of the existing CHIS system to improve community health programs toward universal coverage of PHC is on the prime agenda of the government of Ethiopia [43, 44]. Moreover, digitization is a great enabler to meet the health targets of sustainable development goals and universal health coverage [45].

The government of Ethiopia identified the digitization of CHIS as a critical component of the IR agenda [9]. The initiative is to digitize the CHIS for better data, better information, and better decision making for better health of the community. Thus, the federal Ministry of Health (FMoH) has an intention to demonstrate digitizing CHIS  and make fully scale-up for health data quality, use, and service provision. Moreover, digitization has to replace the traditional approaches of HEWs’ so that they capture information, store data, and provide service in real time using the eCHIS platform.

Though health system digitization brings large benefits to the healthcare delivery system, it has been constrained by a multiple barriers such as the resistance of the health workforce [46], low education [47], poor electricity and network [47, 48], challenges related to system integration [49], poor users coordination [46], limited leaders engagement [50], low users acceptance [50], limited mobile access [48], lack of digital literacy [47] and self-efficacy [51], low interoperability compliance [51], and the need for extensive training during digitization [52]. Moreover, the implementation is likely to be challenged by a number of barriers, as the implementation of a new system is usually constrained by contextual factors. Despite the fact that digitization has sought a lot of attentions, its implementation success has not been high enough for healthcare systems to realize all of its advantages [53].

According to the consolidated framework for implementation research (CFIR), the success or failure of an implementation depends on the intervention’s characteristics [54], outer settings [55], inner settings [54], individuals characteristics [56], and the implementation process [55].

Thus, we explored individual and contextual level enablers and barriers determining eCHIS implementation in light of the CFIR implementation framework.

## Methods

### Study design and participants

An exploratory qualitative study was applied. In-depth interview and a key informant interview were employed to investigate the enablers and barriers to eCHIS implementation. The data were collected from May to June 2022 after one year the intervention has been implemented. The Amhara regional state health bureau (ARHB) along with the University of Gondar (UoG) was responsible for implementing the intervention in the implementation district. Of the 18 interviewees, 12 were male, and their ages ranged from 24 to 46 years. The minimum duration of the interview was 54 min and 28 sec, and the maximum was 2 h, 16 min, and 41 sec. The participants’ work experiences ranged from one to nineteen years (Supplementary table 1).

### Study setting, participants, and sampling

The current research was carried out considering the demonstration site of eCHIS in Wogera district. In this study, the participants were recruited from the implementation district, UoG, ARHB, and the FMoH. Eighteen users and key informants were involved from different positions purposively considering the exposure of participants to the program and their understanding of barriers and enablers of the implementation. The total number of participants was determined according to information saturation where the idea of participants became redundant or no new and relevant information emerged.

### Characteristics and data collection

In-depth interviews were conducted using questions adapted from the CFIR framework for implementation research consisting of five themes [54]. The interview guideline was developed in the local language (Amharic) and pre-tested. In order to ensure its trustworthiness, the guide was reviewed by experts so as to capture the intended data, avoid ambiguity, leading, and sensitive questions. The interviewees were informed about the procedure and then asked to respond to questions related to the enablers and barriers of the eCHIS implementation. Data were collected by the principal investigator, who has a specialty in public health and working experience in implementation research and collecting qualitative data. The interviews were conducted face-to-face in a separate room to ensure data quality and confidentiality and to comfort the respondents if sensitive questions might be raised during the interview. All interviewees were encouraged to discuss their opinions openly regarding the questions forwarded. During each interview, the discussion continued until the information became redundant and no new or relevant data could be obtained. All the interviews were tape-recorded and the back up was maintained daily.

### Data processing and analysis

The data were transcribed into text and translated into English before being coded and grouped into themes and sub-themes with the Nvivo 20 software. A thematic content analysis was conducted based on the reported themes and sub-themes. Each phrase or statement was categorized according to its respective themes and sub-themes. Therefore, the codes, themes, and sub-themes were classified, summarized, and interpreted accordingly. In the results section, the categories of themes and subthemes were supplemented by quotations or verbatim statements of the respondents so as to help the reader better understand the facilitators and barriers to the program. The CFIR framework for implementation research was used for analysis [54]. Deductive [57] and descriptive [42] approaches were used to summarize the data. The final transcription was reread several times to become familiar with the content and compare it with field notes. The related data was grouped under pre-existing CFIR domains and sub-domains [54]. Key phrases or statements were coded under the sub-themes to get more insight into the domains. Finally, quotes that best describe the main themes and sub-themes were presented after describing the findings.

### Ethical considerations

Study approval and ethical clearance were obtained from the University of Gondar ethical review board (R.NO. V/P/RCS/05/2020) and the ethical review committee of the Amhara regional health bureau's research and technology transfer office.

Informed consent was obtained from each study participant. All data were collected based on codes instead of mentioning the names of the respondents to avoid indication of any personal characteristics. The data were secured in University repository and prevented from any access to an unauthorized person. All methods were carried out in accordance with relevant guidelines and regulations.

## Results

The study found that the implementers have an interest in using eCHIS as it helps them produce quality health data, use, and service provision. According to the study, eCHIS implementation was challenged due to outer and inner factors such as the presence of competing projects like CBHI and the lack of institutionalization of eCHIS. Moreover, the implementation was constrained by the limited digital literacy of the implementers and a lack of a defined plan in relation to eCHIS implementation. The barriers and facilitators found in the study themes adapted from the CFIR framework for implementation research were presented in (Table 1).

**Table 1:** Summary of themes and sub-themes highlighting enablers and barriers of eCHIS implementation

CFIR domain and constructs	Themes	Sub-themes(enablers)	Sub-themes(barriers)
**Intervention characteristics** • Intervention source • Evidence strength and quality • Relative advantage • IT infrastructure • Design quality and packaging • Cost	Design, network and electricity, digital literacy and adaptability of the intervention	• Homegrown technology • Considers PHCUs workflows • Designed for data quality and use • Presence of embedded medical eligibility criteria • Reminds service components • Presence of multimedia for service provision • Case validation	• Technology fear • Limited awareness • Limited or absent electricity • Limited or absent network • Unintegration of eCHIS with DHIS2 • Existence of side-by-side manual work
	Data quality and accessibility	• Perceptions of performance expectancy • Ease of use of the system • Implementers eagerness to use • Data protection and accessibility	• Workload of the implementers
	Costs for support and mentoring	• Presence of tablet usage guideline	• Unspecified budget
**Outer setting** • Facility needs and resources • Organizational networks • Peer pressure • External policy and incentives	Commitment to data quality and use Networking and competing interest Policy, procedures and working environment	• eCHIS is flagship agenda of the government • Need to improve healthcare data quality and use • Presence of communication • Establishing of new awarding committee	• Staff turnover • Lack of conducive working environment • Limited networking with external institutions • Presence of competing projects like CBHI • Lack of incentive mechanisms
**Inner setting** • Knowledge and information management • Networks and communications • Tension for change • Gender considerations • Data quality and access • Organizational incentives and rewards • Goals and feedback • Leadership Engagement • Available resources • Access to knowledge and information	Governance and ownership Resource availability and standardization Data quality,use and perception Feedback and regular meetings, and communication Embedded knowledge, skill and attitudes towards eCHIS Motivation and security issue	• Presence of regular PMT meetings • Technical support • Strong need to improve data quality and use • Good perception • Positive attitude towards effort expectancy • Presence of feedback and communication • Mentoring and support from the local and University technical team support • Skill oriented trainings provided • Knowledge shared during training • Self-motivation by using the system	• Absence of help desk for HEWs at district • Low tablet performance (old tablets with low storage) • Lack of specific budget for eCHIS • Lack of institutionalization • Considering eCHIS as extra job • Leaders’ limited comittemnt • Limited local adminstrators engagement • Limited support due to resource • Need of ongoing capacity building • Absence of reward or in kind or financial incentive • Security issue (fear of robbery or tablet lost)
**Individuals’ characteristics** -Knowledge and beliefs about the intervention -Self-efficacy	Trainings, capacity building, and equipping implementers Sociodemographic characteristics Local supports	• HEWs interest to use eCHIS • Presence of active HEWs	• Need of extra trainings and skills • Using tablet for personal use or social events like weeding • Limited self-expectancy • Low educational background • Older age • Absence of experience sharing • Lack of peer-to-peer support • Absence of guideline for reference • Lack of accountable person at district level
**Implementation process** -Planning -Engagement -Reflecting and evaluating	Planning, monitoring, and evaluation Community awareness and engagement	• Engagement of UoG and ARHB • Monitoring the progress, evaluating and giving feedback	• Lack of defined plan • Lack of clear job description for HITs • Lack of community awareness • Absence of local security support • Limited involvement of volunteers and kebele administration • Limited attention at PMT meetings or facility meetings towards eCHIS

**Fig. 1:**
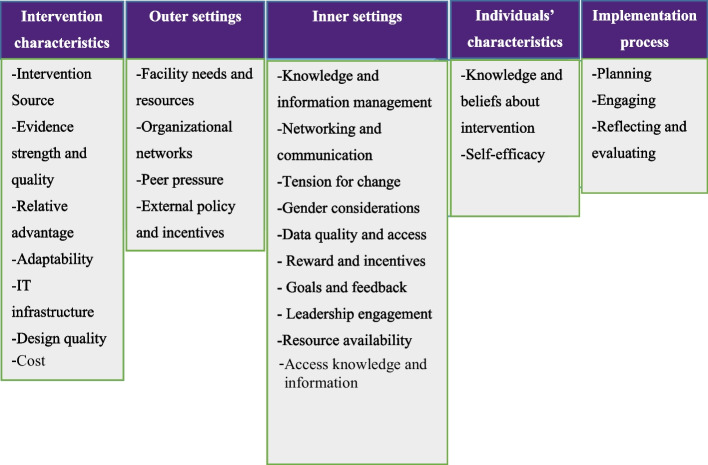
Consolidated framework for implementation research (CFIR)-adapted from Damschroder et al. [54]. Theme 1: Intervention characteristics

Our findings indicated that the implementers well received eCHIS implementation, and they were motivated to use the system for healthcare data capturing and service provision. The training, technical support, and overall benefits of using eCHIS as compared to manual CHIS facilitated the implemeantion. However, some barriers constrained the implementation. The key enablers and barriers that constrained the implementation were discussed using the CFIR framework, and the results were thematized as intervention characteristics, outer settings, inner settings, individual characteristics, and implementation process (Fig. 1).

### Theme 1: Intervention characteristics

#### Intervention source

The perception of key stakeholders about the source of the intervention influenced the implementation. The finding showed that the source being developed by professionals at the MoH level is considered as enabler. As one of the senior respondents (Interview # 5, woman), "eCHIS is developed from scratch by gathering requirements in the context of a lower healthcare delivery system. It considers the workflow of the lower health system."

#### Evidence strength and quality

eCHIS enabled users to organize healthcare data and report timely and provide quality health services. A respondent (Interview # 6, man) explained, “It is helpful for implementers to to use eCHIS as they can easily fill the household information. It provides them clinical decision support during service delivery.” A respondent (Interview #13, woman) added "eCHIS reduces false reports, detects the defaults from service since it shows red pops up. For example, in family planning, it appears in different colors for services that clients expected to receive."

#### Relative advantage

The implementers realized that eCHIS improved their performance and efficiency. A respondent (Interview # 3, man) described, "Using eCHIS does not consume time as they can access information easily." The implementers justified that using the system allows them easily access data and prevents its loss. In this context, a respondent (Interview # 3, man) explained, "I have witnessed that HEWs were happy about using eCHIS, as it is helpful and prevents data loss." According to a respondent, eCHIS has enhanced the quality of health data production at a lower level. A respondent (Interview # 15, woman) confirmed this finding, "eCHIS improved data quality because it reduces false reports as it has validation to allow or refute attributes during data entry."

#### Adaptability

Implementers initially faced technology-related fears but eventually they became familiar. One of the respondents (Interview # 11, man) justified as, "At the beginning, we were a bit confused with technology fear, but through time we were able to use it independently." The importance of creating awareness is vital to adapting and tailoring the intervention. One of the respondents (Interview # 2, man) explained, "System awareness must come first. Implementers and others should be awared about the system."

#### IT infrastructure

The provision of technical support and tablet usage guideline enabled the implementation. A respondent (Interview # 7, man) justified as, "The support and tablet usage guideline helped the users to easily familiarize with the system." The internet and electricity conditions challenged the implementation. A participant (Interview # 1, man) explained as, "…About eight kebeles have no good network, so HEWs synchronize the data by climbing mountains or when they visit town at the weekend." A respondent (Interview # 12, woman) reaffirmed the finding by saying, "I am happy to use it since it eases the job at the health post, but it delays my work because when I finish and synch the data, it takes much time to finish. Even sometimes the network does not work.”

The workload of HEWs challenged the implementation. A respondent (Interview # 3, man) substantiated the idea by saying, "HEWs sometimes feel bored, which is caused by a high workload." Lack of competency was mentioned as a challenge; a respondent (Interview # 3, man) described, “There are HEWs who cannot properly read files written in English. They need local language instead.”

#### Design quality and packaging

The implementers have an interest in using eCHIS and feel that it makes them efficient. According to a respondent (Interview # 7, man), “A multimedia environment existed in eCHIS enabled HEWs to provide quality healthcare. The eCHIS has embedded medical eligibility criteria in visual and audio form for service provision, which facilitated the implementation." Moreover, eCHIS enabled HEWs to promote health information by linking women in the community. A respondent (Interview #12, woman) described as, "One to five and one to thirty that we found at eCHIS is a good package that helped us to arrange and link women to health centers and access health services."

The existence of various reporting forms and the lack of all reportable data elements in eCHIS challenged the implementation. A respondent (Interview # 3, man) described as, "eCHIS does not contain all the components of the manual CHIS." Moreover, eCHIS is not linked with DHIS2. Though eCHIS has made implementers efficient in their work, unintegration of eCHIS with nationally usable system challenged the implementation. One of the respondents (Interview # 7, man) stressed by saying, "eCHIS is not linked with the nationally implemented DHIS2 system. HEWs print out the report from eCHIS and then enter it into DHIS2 for reporting since the data at eCHIS does not directly export to DHIS2."

#### Cost

The finding indicated that the budget required for beginning and ongoing for the implementation is not specified. One of the respondents (Interview # 3, man) justified as, “We have no budget to evaluate performances of the implementers. The program has no specific budget, even we cannot replace a SIM card or a tablet when it is lost or disabled.”

### Theme 2: The outer setting

#### Facility needs and resources

The digitization of the CHIS is currently a flagship initiative of the government. A respondent (Interview # 3, man) described the use of eCHIS in improving health data management practice, "eCHIS is a good platform to realize the IR agenda in terms of improving data quality and information use." A respondent (Interview#13, woman) added, *"*It fulfills our need since CHIS takes much time to finish activities using manual tools. However, eCHIS saves our time and improves quality data production and service provision."

The culture of retaining trained individuals is limited. One of the respondents (Interview # 4, man) explained, "The new individuals faced difficulties to handle the system due to staff turnover." The lack of a conducive working environment constrained the implementation. A respondent (Interview # 3, man) justified by saying, "There were no shelves and tickler box in some health posts. HEWs were using the floor as the store for registers, reports, and tally sheets.”

#### Organizational networks

The finding indicated that interfacility networking enabled the implementation. A respondent (Interview #13, woman) mentioned, “We have a network with our nearby health center and communicate about the system’s usability. The district gives feedback to health center and and health center to health posts in structured way and it gave motivation and enabled us to complete household registration in a short period of time.”

The findings showed that formal and informal networking needs emphasis for a better connection. In this regard, a respondent (Interview # 18, man) explained, "The UoG technical team has a network with the woreda health office, the ARHB, and the MoH team. ARHB and other concerned organizations should take responsibility to realize a better implementation."

#### Peer pressure

According to the current study, there was a lack of leaders’ willingness to conduct eCHIS side by side with other competing activities. A respondent (Interview # 8, man) confirmed by saying, “…For example, during a campaign, it would have been a good opportunity to link eCHIS activities to run together, but leaders at lower levels were not willing to allow.” HEWs were facing difficulties remembering their tablets’ PIN codes or passwords after a long stay at campaign. Some of them forget how to open and close the system. A respondent (Interview # 3, man) reassured, "Some of the urgent activities that often challenge HEWs in implementing eCHIS include community-based health insurance. HEWs stay for a long period in the community for campaign activities.” Moreover, a respondent (Interview #15, woman) added how competing demands have influenced the implementation by saying, “Campaign activities were affecting our motivation to use eCHIS properly. For example, if the district receives direction for a given campaign from a higher level, all the efforts of HEWs are expected to engage in it."

#### External policy and incentives

Lack of incentive mechanisms was described as a factor influencing the implementation. A respondent (Interview # 4, man) described as, "Whether you work hard or not, there is no recognition. I think this should be changed." Another respondent (Interview # 9, man) emphasized, "We do not have rewarding mechanisms for the best performers in our facility, but now we have created a committee this year to screen and reward those who perform better in all the programs." Moreover, a respondent (Interview #13, woman) added her feeling, “We need recognition from the district office and other responsible bodies. Thank you by itself is a great thing and motivates us to work better.”

### Theme 3: Inner setting

#### Knowledge and information management

The interviewees described the current PHCUs structure as an advantage, allowing for easy implementation. In this regard, a respondent (Interview # 18, man) forwarded his opinion, “We provide biweekly support to facilitate the implementation and have a Telegram channel to communicate with implementers." HEWs have different skill and knowledge and require assistance and experience sharing. A respondent (Interview # 18, man) mentioned how sharing experience is vital to get knowledge and achieve the intended goal, "The implementers have different understanding and skill levels. They need continuous support and experience-sharing since it is important to gain knowledge and understand the drawbacks of the implementation." It was argued that eCHIS has received little attention at PMT meetings. A respondent (Interview # 1, man) explained, "The attention given for eCHIS is limited. They do not consider it in their weekly or monthly assessment plan as they do for data verification, completeness, and timeliness." He added, "The problem is that we consider eCHIS as extra work. The implementation must be reviewed during PMT meetings." Moreover, a respondent (Interview #16, woman) added how distant health posts influenced close monitoring and feedback during implementation by saying, *"*When we send feedback to health centers, it will motivate them, but some health posts are located too far from the district office to monitor the implementation." For example, those health posts in remote areas or kolla zuriya are difficult to monitor as intended."

#### Networking and communications

Networking and communication are mentioned as facilitators. A respondent (Interview # 3, man) explained his feeling, "At woreda level, there is a feedback chain. The woreda announces the name of health posts that submit or fail to submit data on time and ranks them accordingly." In this regard, another respondent (Interview # 10, man) said, "The woreda Health Office provides feedback weekly to the health center. We provide feedback to HEWs and evaluate their performance." It has been discussed that the implementers need help desk availability. Supporting this finding, a respondent (Interview # 6, man) explained his feelings as, “At the FMoH level, we have a help desk where two individuals are assigned to respond to eCHIS-related questions. However, HEWs were not able to get such kind of help at district level."

#### Tension for change

The government of Ethiopia launched eCHIS in the lower health system to improve health data. Interviewees reported that the implementation at healthcare facilities assisted them in managing health data. One of the respondents (Interview # 3, man) mentioned, "eCHIS helped the implementers to improve data quality and information use."

A respondent (Interview #12, woman) added how data quality improvement is a needy and eCHIS has ability to help them by saying, “I am happy to use eCHIS since it gives us quality data. Thus why we did campaign to convert CHIS into eCHIS in a short period of time.”

#### Gender considerations

It has been mentioned that eCHIS has many benefits over CHIS. A respondent (Interview # 7, man) explained,"eCHIS helps implementers collect and report information timely. It helps them to improve their performance and is suitable to digitize CHIS." The implementers fear moving from place to place alone while holding the tablet in the community, especially during house-to-house visits. According to one respondent (Interview # 2, man), "They have a fear of walking alone because of the distance they cover and the fear of losing the tablet." It has been argued that a multidisciplinary approach could help them. A respondent (Interview # 3, man) justified his feeling, "At national level, the female-based HEP should be revised to include males and females from different fields of study." Another respondent (Interview # 4, man) added, "HEWs who have kids carry the registers along with their kids while they do house-to-house registration. Secondly, they have a fear to walking alone because of the distance they cover."

#### Data quality and access

The implementers perceived that eCHIS helped them directly or indirectly manage HPs documents easily, which is exhaustive for manual data handling. One of the respondents (Interview # 13, woman) justified, "House-to-house registration is easy when we use a tablet because we can cover all households expected within a day and reduce errors. Sometimes, we conduct HP activities at night using the tablet." Using eCHIS provides safe resources and protects data from natural and human-made factors and increases data accessibility. A respondent (Interview # 3, man) explained the idea in this way, "eCHIS prevents data loss when natural disasters or man-made factors occur. Thus, eCHIS is exempt from this kind of harm and data can be protected easily."

#### Organizational incentives and rewards

Though motivation is an important element to boost the mental component of the implementers, there are no formal and/or informal motivation mechanisms. A respondent (Interview # 14, woman) expressed her feelings by saying, "Those who work better and recognized will continue to do good work. Others who missed the recognition due to their low performance may try to become awardees in the next round." She added, emphasizing the idea, "For example, our HP is a model in eCHIS implementation, but we did not have any incentive or reward. None of us have received recognition. We did not even get a thank you or a certificate for our good work."

#### Goals and feedback

The interviewees discussed the value of feedback. One of the respondents (Interview # 3, man) expressed his feeling, "During my field visits, I have come to observe that feedback is given on a weekly basis, and there has been improvement." The study identified that, though there is recognition of the feedback provided, there are still areas for improvement. A respondent (Interview # 6, man) described, "The feedback mechanism is not as strong as it should be. For example, close supervision and feedback mechanisms were intermittent from higher officials. It is good if the feedback system of the dashboard is completed to receive reports and provide feedback in real-time." Another respondent (Interview #14, woman) explained the finding by saying, "Even feedback is only requested by the planning officer. If he becomes busy, we delay the reports. If no one is concerned or asks us about the feedback and activities report, we will not be initiated by ourselves."

#### Leadership engagement

Many participants expressed that the commitment of healthcare leaders was limited. The finding indicated that they give priority to directions given from higher officials. Emphasizing this idea, a respondent (Interview # 4, man) mentioned, "The health insurance has the administrators' attention, so they expect us to focus on the health insurance program." Another respondent (Interview # 18, man) added,"eCHIS is not a program to evaluate leaders as CBHI." Participants described that eCHIS is one of the government's flagship agenda, but leadership engagement and ownership are limited at the lower levels. Supporting this idea, a respondent (Interview # 6, man) explained, "People take eCHIS as part of the duties of planning officers and/or HITs. They are not committed to eCHIS because they did not understand eCHIS as it is a big governmental initiative.” A respondent (Interview #13, woman) added,“We are eager to use eCHIS but the commitment given from leaders is not attractive and motivational.”

#### Available resources

The unavailability of resources constrained the the implementation. Supporting this idea, a respondent (Interview # 1, man) expressed, "As there is no budget for eCHIS support, we use the annual budget to support eCHIS." The current findings also indicated that infrastructure and resource constraints were commonly shared challenges. There are HEWs who perform their activities at home due to the absence of HPs at their kebele. Emphasizing the finding, a respondent (Interview # 3, man) described, "There are HPs that have no space to store documents. Some of the HEWs keep the documents at home.” It was widely argued that the speed of the tablets was challenging. A respondent (Interview # 5, woman) explained, "The performance of the current tablets is challenging since they have low capacity and do not allow us to execute eCHIS activities as needed.”

#### Access to knowledge and information

The finding indicated that the implementers were pleased with the training provided and the technical support provided by UoG and HIT professionals designated at the woreda level. We discovered that implementers expressed willingness to ongoing training and capacity building. One of the respondents (Interview # 7, man) described, "HEWs require assistance with technology implementation because they have studied health sciences, and it is important to support them."

However, implementers need frequent updates regarding knowledge and skills related to eCHIS. A respondent (Interview #14, woman) expressed her feeling as, "It has been a long time since we have been detached from education. It is difficult to write clients’ names and Gott’s or Kebele’s names properly. Using English to work is a headache for us; for example, some HEWs even donot write their names properly. We need all things in eCHIS to be in local language to facilitate working activities."

### Theme 4: Individual characteristics

#### Knowledge and beliefs

Our study revealed that implementers who received basic eCHIS training were motivated to use eCHIS, and perceived that it would increase their performance and efficiency. A respondent (Interview # 3, man) expressed, "HEWs are happy about using eCHIS. I have met a HEW who use eCHIS, she is happy to use it. For example, if she has to visit 10 households, she should have to have 10 folders in the manual case, yet using a tablet reduces such a burden." According to the current study, tablets are being used for unintended purposes. A respondent (Interview # 4, man) reaffirmed, "HEWs install different applications to play files such as videos, music, photos, and other applications. They give the tablet to their husbands, friends, and relatives. I encountered a person who came to me holding HEW’s tablet and asked me to install a game.” Another respondent (Interview # 3, man) emphasized the idea, "As they store more personal files in the tablets, there will be less storage space left for eCHIS activities.” We noticed that capacity building and support are important components to fill the gaps among implementers. A respondent (Interview # 4, man) explained, "If they face any problem related to tablet usage, they call the woreda office and/or UoG supporting team.”

#### Self-efficacy

The finding showed that there are implementers who can use the system by themselves. One of the respondents (Interview # 4, man) justified, "There are competent implementers who can understand it fast and get into work easily." A respondent (Interview # 3, man) added, "There are implementers who can understand how the system works and can manage the network problem by themselves.” However, there are some implementers who need support due to their limited self-expectancy. A respondent (Interview # 18, man) expressed, "As to me, there should be peer-to-peer cooperation. In one HC, there are active HEWs, and it is better to choose them as a leader and connect them in a group." The study indicated that older age and low educational readiness constrained the implementation. A respondent (Interview # 2, man) described the above finding, "Honestly speaking, there are HEWs who cannot even arrange and register data properly. Most of the time, those who encounter problems are because of their older age and low education status." Another respondent (Interview #16, woman) explained that some HEWs have a fear of using technology by saying,* "*Some HEWs are not willing to use the tablet because of their limited knowledge and skill on smart phone usage and they feel that if they touch the tablet, everything within it would be damaged."

A respondent (Interview #14, woman) mentioned that supporting and continuous mentoring are vital to implementers, "We got support and mentorship from a local mentor. Some HEWs need local mentors and help easily when they face challenges."

The participants mentioned that working manuals would be better to fill the knowledge gap. A respondent (Interview # 5, man) expressed, "Availing the eCHIS manual at the community level or integrating guidelines or video tutorials to eCHIS using local language could help to fill the gap and reduces waiting time for them."

### Theme 5: process

#### Planning

It was mentioned that lack of a defined schedule for activities, budget allocation, job description, and roles and responsibilities of individuals were challenged the implementation. A respondent (Interview # 6, man) described his feeling, “…In eCHIS there is no clear integrated refreshment training and other related plans.” It has been revealed that there must be an accountable person for the program. One of the respondents (Interview # 3, man) explained, “The eCHIS should be incorporated in the annual plan. There should be a person who is accountable to assist the implementation.” Another respondent (Interview #16, woman) added the finding, saying, "We do not have a clear plan for using eCHIS. We do not have a specific budget. Some HEWs make the tablets much busier with personal and emergency activities like CBHI due to a lack of a defined and clear plan in advance."

#### Engaging

It has been said that leaders engagement was limited and challenged the implementation. A respondent (Interview #14, woman) explained as, “Leaders were not engaged to support eCHIS activities as intended.” It was mentioned that the UoG technical team and ARHB played a role as an external change agent supporting the implementation. A respondent (Interview # 1, man) explained, “We got assistance from the region and UoG. This created a good opportunity to implement the intervention in a better way.” However, kebele leaders, volunteers, women’s development army, community, and religious leaders’ engagement should be emphasized for the betterment of the implementation. A respondent (Interview # 18, man) explained his feeling, saying, “Apart from health sector leaders, kebele administration and volunteers should participate in the implementation since it is a multi-sectoral program. It should not be only the health sector leaders’ role.”

#### Reflecting and evaluating

Though there is a common platform to monitor the progress of eCHIS using telegram and technical working groups, there is a gap in monitoring, evaluating, and giving both qualitative and quantitative feedback. A respondent (Interview # 8, man) described, “The challenge is at woreda level. There is a PMT team to assess the performance of key indicators at facility level, but the attention given to eCHIS is limited.” A respondent (Interview #16, woman) expressed her feeling that evaluation and monitoring need emphasis at the district level, saying, "Leaders are busy with emergency and campaign activities; they do not give time to monitor and evaluate eCHIS activities as intended, and it should not be only a duty of a planning officer." Moreover, another respondent (Interview #14, woman) added, "Only one individual is concerned about eCHIS at the woreda level to follow the progress. What if all leaders get concerned regarding eCHIS? This culture should be changed. We wish health system leaders would actively monitor and evaluate eCHIS activity regularly."

## Discussion

The aim of this study was to explore the factors influencing eCHIS implementation. Accordingly, intervention characteristics, outer setting, inner setting, individuals’ characteristics, and implementation processinfluenced the eCHIS implementation.

The positive perception towards eCHIS usage might be due to quality health data production, ease of use, reduced errors, data protection, and increased accessibility. The WHO recommends CHWs to use digital tool [27]. A study showed that community health digitization using mobile applications supports the services delivered by CHWs [58].

The current study considered the existing context and interoperability and used local language that motivated implementers to adapt and use the system easily. The perception of key stakeholders about the intervention source influenced the implementation [59]. Availing data at higher levels could enhance data use practices. It is believed that a health system supported by technology brings change to the health system. Studies show that digitization of healthcare data impacted healthcare and health outcomes [21–23, 60–63] and improves productivity and efficiency of health staff [64].

The finding indicated that using eCHIS is considerably important to produce quality data, reduce workload, and maintain data security. The level of users’ perception of the advantage of implementing the intervention matters its implementation success as compared to the existing approach [65]. Digital health solutions enable CHWs to generate quality health data [30], improve healthcare delivery [25], and be more effective in their job-aids [66, 67]. Likewise, it creates enjoyment [31] and benefits them to keep data from human and natural factors that could damage the data.

Moreover, this study is part of a big project in which the authors have conducted a quantitative study to measure the impact of eCHIS implementation on maternal health service coverage [68]. The study showed that in the pre-post comparison, 44(10.6%) and 135(32.5%) of women were retained fully on the maternal continuum of care. Women in the eCHIS intervention group had a higher chance of completing the maternal continuum of care than women in the comparator group, i.e., one in every five women completes the maternal continuum of care at baseline. In comparison, one in every three women completes the maternal continuum of care at the study endpoint in the intervention district. Thus, the study found that the eCHIS intervention had impacted the improvement of the maternal continuum of care in rural northwest Ethiopia. The findings agreed with past studies showing that using mobile health involving community health workers has impacted maternal healthcare utilization [25, 69]. The possible reason for the improvement of the maternal continuum of care could be that a digitized community health information system could enable HEWs to provide maternal health services and link pregnant women with health facilities. According to the literature, mobile health interventions have a positive effect on antenatal care, skilled birth attendance, and postnatal care improvement [70], and empowering HEWs [71] yields improved maternal health service utilization [69].

The limited attention of leaders in our study might be due to, most of the time, they give emphasis to directions given from higher level. A study showed that political support is vital to HIS digitization [50]. Creating ownership and awareness among leaders is vital to realizing community health digitization. HEWs are overwhelmed with multiple community activities, [12]. The increased workload of HEWs could affect the digitization of community health program. Moreover, our study revealed that HEWs’ competency influenced the implementation. A study showed that low education level influences HIS digitization [47] which could be due to the low educational readiness and digital literacy among HEWs [16]. Thus, continuous mentorship and capacity building are recommended to equip implementers to use technology [72], as technology implementation requires continuous monitoring, learning, and adaptation at the initial stage [24]. The chance of getting adequate internet in rural areas is limited and found to be a challenge to synchronize data into a central server since the coverage of the network and electricity are poor or absent in rural areas [73]. FMoH should emphasize collaboration with other sectors to balance electricity and internet distribution. Studies indicated that electricity and network accessibility are challenging factors in digitization [47, 48].

In eCHIS, the system has a multimedia feature however lacks integration with DHIS2. A study showed that an intervention's quality design and package affect the implementers' perception [74].

For the betterment of the implementation, the cost should be specified at lower health system. An appropriate cost breakdown such as beginning costs progress costs should be considered [54]. The government of Ethiopia has been investing about $US 5,073,996.00 each year on a manual HMIS despite poor data quality [75]. Digitization of the CHIS guarantees the quality of health data production, use, and service provision.

The findings in the current study indicated that eCHIS enabled HEWs to capture and report data using an electronic platform easily.

The Ethiopian government has strong desire to transform data management practice and training HITs as a focal of HMIS units [76], however, there is a turnover of trained professionals. The reason could be absence of HIT retention mechanisms. The technology implementation requires competent personnel [77]. The incentive mechanism should be strengthened. Identifying and prioritizing the need as well as barriers and facilitators should be a prime focus to meet the goal of community health digitization [78]. A study in Ethiopia shows that staff motivation is a remedy to good data management and quality data production [17].

It has been widely discussed that UoG, RHB, and MoH supported the implementers. However, there was a gap in networking people in the community. The WDA is known in the community for reminding pregnant women for maternity care and linking them to a health facility [79]. Adapting digital health solutions in a contextual environment could bring challenges and require the strong commitment of the government, stakeholders, volunteers, and people in the health system.

We found that the campaign activities influenced the implementation, as mimetic projects hinder implementation success [54]. According to a study, the CHWs are loaded with many activities [12] Sometimes, they may be forced to engage in accidental activities for a long period of time which could affect community health digitization. There is no external incentive rules regarding eCHIS implementation. Incentivization for better performance has a motivational component that could encourage implementers to work in a better way. Multiple studies suggest that incentivization brings improvement in the healthcare system [80, 81].

We found that eCHIS is not discussed during performance monitoring team as intended. The less emphasis might be due to the lack of institutionalization of eCHIS at health facilities. We believe that institutionalization facilitates CHIS digitization. The current study identified biweekly support, supervision, technical team assistance, and monthly meetings enabled the implementation. However, the unavailability of the help desk at PHCUs constrained the implementation. The emphasis should be given to establishing a help center at the district level, giving close support and timely feedback, and maintaining community mobilization.

The finding indicated that implementers perceived the implementation as needing change for improved health data use and service provision. Digitization of the HIS and promotion of data use were the major pillars of the HSTP in Ethiopia [16]. The reason for intolerance of change in using eCHIS could be that the digitization of CHIS facilitates data capturing, storage, transmission, and retrieval, saves lives, and improves the healthcare system. Digitization of CHS enhances health sectors and helps achieve the goal of the IR. The eCHIS allows electronic sharing of household and individual information between HEWs and other health staff that enables them to easily monitor and update information [24].

The finding showed that HEWS hesitate to move from place to place due to fear of tablet theft or robbery. It is recommended to strengthen the HEP program by allowing all gender and professional mix approach.

The absence of a formally delegated person and a dedicated budget to eCHIS-related activities constrained the implementation.

The feedback from a higher level has a valuable meaning for health workers, and attention should be given to providing timely feedback. It is vital to clearly communicate, act on goals, and give feedback. A study showed that poor feedback contributed to poor health data generation and use [15].

The limited leaders’ engagement in the current study might be due to lack of awareness toward eCHIS. Studies showed that weak and limited leadership engagement [82] and a lack of coordinated actions [77] hinder effective implementation. The linkage of eCHIS with health system structure and engaging leaders in eCHIS implementation is vital. Leadership engagement and support for the healthcare industry in all the processes of healthcare could be a remedy to treat anomalies in the health system [83].

We found that there is a lack of resources for eCHIS implementation at the woreda level as dedicated budget for its implemenation. Studies show that challenges during the implementation of digital health solutions, such as the initial and ongoing capacity building trainings [84], poor network and difficulties in phone charging [85], low resource [84], unreliability or absence electricity and network [47, 48] could hinder the implementation.

The skills and knowledge of HEWs vary from one to another and requires mentoring and supporting. Studies showed that inability to use the system could affect the implementation(26), and intensive training with continuous refreshment could help them realize digital community health information system [72].

Furthermore, the finding showed that implementers misuse the tablet which depletes its storage. Knowledge and beliefs of the implementers toward intervention are vital tools [86]. Moreover, the tablet usage guideline helps to restrict tablet usage for intended activities.

We found that there is limited self-efficacy. It is the implementer’s belief in their own capabilities to execute courses of action that help them achieve the implementation goals [87]. The reason could be due to the lower educational readiness of HEWs. A study showed that computer literacy is one of the main factors affecting users acceptance of technology use [39]. The WHO recommends CHWs use digital technology [27] to support CHWs by enabling them through capacity building [72]. The possible reason might be that those who had self-expectancy could not face difficulty adapting the emerging technology to community level data management and service provision. The current evidence in the feasibility and effectiveness study on digital health indicated that the level of computer literacy had influenced digital health implementation among community health workers [84].

The finding also indicated that implementers in older age faced difficulty coping with the system or a challenge in familiarizing themselves with the technology. The reason could be the older implementers lack concentration and have little chance of acquainting themselves with the smart phone, and they may encounter technology phobia. In addition, they may have less exposure to digital technology and be less comfortable adopting and using technology [39], as their older age appears to be a barrier due to their anxiety (fear of technology use) [20, 38]. On the contrary, younger age is the common individual enabler for digital health implementation [32]. The reason could be the younger age might have interest to use, perceive the system can save time and help them to share knowledge [38]. Moreover, a study confirms that individuals who are in the young age group [32] and have a good educational level are enablers to adopt healthcare digitization [32].

Though the government and supporting partners envisioned community health digitization, the lower level lacks a defined plan for eCHIS implementation. A study showed that low financial investment [84] affects performance of the program. Involving appropriate individuals and organizations is vital for successful implementation [87]. The current finding showed there is limited awareness creation among people in the lower health system. The study showed that social influence has a positive contribution to technology use [88]. The engagement should include kebele administration, and volunteers in the health system. A study showed that limited administrative engagement contributes to lower performances [82].

The current study showed that monitoring and evaluation from higher level is limited. Provision of regular mentorship, monitoring, and evaluation are vital to consider during implementation as it is a collaborative learning process and provides a sense of modeling, coaching, and self-doing skills. The limitations of our study include the context-specific nature of the findings, which should be considered when applying eCHIS implementation to other areas. The purposive sampling of the key informants and in-depth interviews in the current study may have introduced bias into the findings of this study.

## Conclusion

The current study provides actionable findings on factors that facilitate and constrain the the implementation. The findings can be used to take measure to improve the implementation of the eCHIS. The key factors enabling the implementation were the availability of support team and regular meetings. eCHIS improves performance and efficiency and has ability to create motivation. The facilitators and barriers reported in the current study can be expanded further to improve the eCHIS program in a larger-settings and be used in similar contextual environments. Overarching barriers were the limited leaders' commitment, resource availability, institutionalization, and need of ongoing capacity building. More specific factors for improvement include ensuring network and electricity, community engagement, and enhancing the digital literacy of the implementers. The CFIR framework for implementation research has a broad application. We authors encourage others to use the CFIR framework as it allows researchers to explore enablers and barriers systematically and comprehensively.

## Supplementary Information

**Additional file 1.** 

**Additional file 2.** 

**Additional file 3.**

## Data Availability

All relevant data are within the manuscript. The data upon which these findings were developed can also be available from corresponding author upon request.
