# Supplementary material for: Individual and contextual level enablers and barriers determining electronic community health information system implementation in northwest Ethiopia
Source: BMC Health Serv Res. 2023 Jun 16;23:644. doi: 10.1186/s12913-023-09629-8 (PMC10273575; doi:10.1186/s12913-023-09629-8)
Supplement: Supplementary file 1 — Additional file 1. [file 12913_2023_9629_MOESM1_ESM.docx]

# **Assessment tool for electronic community health information system facilitators and barriers**

# KII In-depth-interview guideline to assess barriers and facilitators of e-CHIS intervention:

Date: ____________________________

Identification (ID) code: _____________________

**Introduction**

Interviewer(s) should introduce him/herself (Name and Organization). Interviewer should introduce the e-CHIS purpose and objective of the interview: We are conducting a study to assess barriers and facilitators of electronic community health information system in Central Gondar zone which will contribute to improve implementation of digital healthcare in the Zone and to effort wider scale up in the country. The purpose of this interview is to explore the opinions of important stakeholders of e-CHIS, such as yourself, to help us develop strategies that will address the facilitators and barriers to successful implementation of electronic community health information system. We plan to conduct at least 26 interviews. The information obtained through this interview will be used for improvement of e-CHIS implementation in the Woreda and in the country as well. We would now like to ask you a few questions about your opinion regarding facilitators and barriers of e-CHIS implementation.

Are you interested to participate? 1) Yes 2) No

1. Facility/organization name_______________ Age_________Sex_________Education level _________Professional experience ____________Responsibility ______________
2. **Intervention characteristics**

Explain the following in terms of facilitators and barriers in eCHIS implementation

- 1. Perception of eCHIS is internally or externally developed
  2. Stakeholders’ perceptions of the quality and validity of evidence supporting the belief that the intervention will have desired outcomes
  3. Stakeholders’ perception of the advantage of implementing the eCHIS versus CHIS
  4. The degree to which eCHIS can be adapted, tailored, refined, or reinvented to meet local needs.
  5. Perceived difficulty of implementation, reflected by duration, scope, radicalness, disruptiveness, centrality, and intricacy and number of steps required to implement
  6. Perceived excellence in how the intervention is bundled, presented, and assembled
  7. Costs of the intervention (beginning) and costs associated with implementing (progress) the intervention including investment, supply, and opportunity costs

1. **Outer setting**

Explain the following in terms of facilitators and barriers in eCHIS implementation

- 1. The extent to which facility needs eCHIS use, as well as barriers and facilitators to meet data quality, use and service provision are accurately known and prioritized by the woreda
  2. The degree to which the woreda health office is networked with other external organizations
  3. The competitive pressure to implement eCHIS (presence of other prioritized agenda or program)
  4. Presence of external strategies to spread eCHIS including policy and regulations

1. **Inner setting**

Explain the following in terms of facilitators and barriers in eCHIS implementation

- 1. Social architecture, age, maturity, and size of an organization in which eCHIS is implementing? (TWG, forum, participants, meting PMT, Role of PPMED), Platform (Telegram), eCHIS center of excellence
  2. The quality of formal and informal communications within an organization about e-CHIS
  3. Perception of data quality, information use, and quality service provision issues needing change
  4. The degree of tangible fit between meaning and values attached to the innovation by involved individuals, how those align with individuals’ own norms, values, and perceived risks and needs, and how the innovation fits with existing workflows and systems
  5. The implementers, facilities and case teams shared perception of the importance of the implementing e-CHIS within the organization
  6. Organizational incentives such as goal-sharing awards, performance reviews, promotions, and raises in salary
  7. The degree to which e-CHIS implementation is clearly communicated, acted upon, and feedback to staff, and alignment of that feedback with its implementation
  8. The commitment, involvement, and accountability of leaders and managers with the implementation
  9. The level of resources dedicated for implementation and on-going operations, including money, training, education, physical space, and time, tablets, budget, server at regional level
  10. Ease of access to digestible information and knowledge about the intervention and how to incorporate it into work tasks.

1. **Characteristics of individuals**

Explain the following in terms of facilitators and barriers in eCHIS implementation

- 1. Individuals’ attitudes toward and value placed on the e-CHIS use as well as familiarity with facts, truths, and principles related to the e-CHIS
  2. Individual belief in their own capabilities to execute courses of action to achieve eCHIS target

1. **Implementation process**

Explain the following in terms of facilitators and barriers in eCHIS implementation

- 1. The degree to which a method of tasks for implementing eCHIS is developed or planned in advance: strategic plan, cost benefit analysis plan, need base plan, defined budget plan, roadmap, complete eCHIS time plan, new tablet replacement plan, feasible plan
  2. The engagement of leaders and other appropriate individuals in the implementation and use of the intervention eCHIS? engagement of regional, zonal, woreda and federal leaders and partners, and stakeholders
  3. The reflecting and evaluating of eCHIS process, mentorship, monitoring, evaluation, action based on evaluation, feedback on eCHIS progress regularly, documentation process, regular tablet and SIM card evaluation, and supervision
