# Supplementary material for: Individual and contextual level enablers and barriers determining electronic community health information system implementation in northwest Ethiopia
Source: BMC Health Serv Res. 2023 Jun 16;23:644. doi: 10.1186/s12913-023-09629-8 (PMC10273575; doi:10.1186/s12913-023-09629-8)
Supplement: Supplementary file 2 — Additional file 2. [file 12913_2023_9629_MOESM2_ESM.docx]

**Supplementary table 1: Socio-demographic characteristics of the study participants, 2022**

| **Participant ID** | **Sex** | **Age in years** | **Educational status** | **Work experience** | **Type of users** |
| --- | --- | --- | --- | --- | --- |
| P1 | Male | 30 | BSC | 10 years | District M and E officer |
| P2 | Male | 34 | BSc | 9 years | Zonal eCHIS focal |
| P3 | Male | 35 | BSC | 10 years | Zonal HMIS focal |
| P4 | Male | 26 | Diploma | 3 years | Facility eCHIS focal |
| P5 | Female | 32 | MSc | 9 years | MoH eCHI focal |
| P6 | Male | 37 | MSc | 13 years | MoH eCHIS specialist |
| P7 | Male | 46 | MPH | 19 years | Regional HMIS focal |
| P8 | Male | 35 | MPH | 11 years | Regional eCHIS focal |
| P9 | Male | 26 | Diploma | 4 years | Facility eCHIS focal |
| P10 | Male | 28 | Diploma | 8 years | Facility eCHIS focal |
| P11 | Male | 30 | Diploma | 5 years | Facility eCHIS focal |
| P12 | Female | 26 | Level IV diploma | 6 years | HEW |
| P13 | Female | 26 | Level IV diploma | 10 years | HEW |
| P14 | Female | 37 | Level IV diploma | 8 years | HEW |
| P15 | Female | 28 | Level IV diploma | 5 years | HEW |
| P16 | Female | 33 | BSc | 14 years | District eCHIS focal |
| P17 | Male | 24 | BSc | 1 year | eCHIS local mentor |
| P18 | Male | 28 | MSc | 7 years | University eCHIS focal |
| P= Participants; BSC= Bachelor of science; MSc=Masters of science; MPH=Masters in public health | | | | |  |
