# Supplementary material for: Individual and contextual level enablers and barriers determining electronic community health information system implementation in northwest Ethiopia
Source: BMC Health Serv Res. 2023 Jun 16;23:644. doi: 10.1186/s12913-023-09629-8 (PMC10273575; doi:10.1186/s12913-023-09629-8)
Supplement: Supplementary file 3 — Additional file 3. [file 12913_2023_9629_MOESM3_ESM.docx]

**Supplementary table 2: Themes, sub-themes and descriptions of the study variables adapted from CFIR framework for implementation research**

| **Themes** | **Sub-themes** | **Description** |
| --- | --- | --- |
| **Intervention characteristics** | Intervention source | Perceive that eCHIS internally or externally developed. |
|  | Evidence strength and quality | Stakeholders’ perceptions of the quality and validity of evidence supporting the belief that the intervention will have desired outcomes. |
|  | Relative advantage | Perception towards advantage of eCHIS as compared to CHIS. |
|  | Adaptability | eCHIS can be tailored, refined or reinvented to meet the issue of data quality, use, and service provision. |
|  | IT-Infrastructure | The challenges in the implementation of eCHIS. |
|  | Design quality and packaging | Perceived excellence in how the intervention is bundled, presented, and assembled. |
|  | Cost | Costs of the intervention (beginning) and costs associated with implementing (progress) the intervention including investment, supply, and opportunity costs. |
| **Outer setting** | Facility needs | Extent of facility needs eCHIS for data quality and use, as well as barriers and facilitators to meet data quality and use, are accurately known and prioritized. |
|  | Organizational networks | The degree which the woreda health office is networked with other external organizations in implementation of eCHIS. |
|  | Peer pressure | Competitive pressure to implement eCHIS (presence of other prioritized agenda or program). |
|  | External policy and incentives | External strategies to spread eCHIS including policy and regulations. |
| **Inner setting** | Knowledge and information management | Social architecture, age, maturity, and size of an organization in which eCHIS is implementing, existing attributes within the organization that enable or hinder the implementation. |
|  | Networks and communications | Formal and informal communications within an organization about eCHIS. |
|  | Tension for change | Perception to data quality and information use and service provision issues or needing change. |
|  | Gender considerations | Is about how the eCHIS fits with existing workflows and systems. |
|  | Data quality and access | Stakeholders’ shared perception of the importance of the implementing eCHIS within the organization |
|  | Organizational incentives and rewards | Organizational incentives such as goal-sharing awards, performance reviews, promotions, and raises in salary. |
|  | Goals and feedback | The degree to which data quality, information use and service provision are clearly communicated, acted upon, and feedback to staff, and alignment of feedback with the targets of the implementation. |
|  | Leadership Engagement | Commitment, involvement, and accountability of leaders and managers with the implementation. |
|  | Available resources | The level of resources dedicated for implementation and on-going operations, including money, training, education, physical space, and time, tablets, budget, server at regional level etc. |
|  | Access to knowledge and information | Ease of access to digestible information and knowledge about the eCHIS and how to incorporate it into work tasks. |
| **Individuals’ characteristics** | Knowledge and beliefs about the intervention | Attitudes toward and value placed on the e-CHIS use as well as familiarity with facts, truths, and principles related to the e-CHIS. |
|  | Self-efficacy | Individual belief in their own capabilities to execute courses of action to achieve eCHIS target |
| **Implementation process** | Planning | Method of tasks for implementing eCHIS is developed or planned in advance. |
|  | Engagement | Engagement of leaders and other appropriate individuals in the implementation and use of the intervention. |
|  | Reflecting and evaluating | Reflecting and evaluating of eCHIS process such as mentorship, monitoring, evaluation, feedback on eCHIS progress, documentation process etc. |
